# Ligament Augmentation and Reconstruction System Failures in Repair of Grade V Acromioclavicular Joint Dislocation

**DOI:** 10.1155/2017/3792610

**Published:** 2017-09-06

**Authors:** Martin K.-H. Li, David Woods

**Affiliations:** Department of Trauma and Orthopaedics, Great Western Hospitals NHS Foundation Trust, Swindon SN3 6BB, UK

## Abstract

The Ligament Augmentation and Reconstruction System® (LARS®) represents a popular synthetic anatomical reduction method for acromioclavicular joint dislocation by means of coracoclavicular ligament reconstruction. To our knowledge, no early failure has been documented in the literature. We present two unusual cases of LARS failure, one at four months after implant and the other at three weeks, without obvious causes, requiring re-do reconstruction, and discuss potential contributory factors.

## 1. Introduction

Anatomical coracoclavicular ligament reconstructions, such as the ligament augmentation and reconstruction system (“LARS comp,” Arc-sur-Tille, Dijon, France), have been popularized in acromioclavicular joint (ACJ) dislocations owing to close biomechanical properties to the native joint [1]. Numerous options exist for anatomical reconstruction, including screws, hook plates, and coracoclavicular reconstruction using free tendon grafts, with differing biomechanics [2, 3], but comparable clinical results [4].

To our knowledge, no trial or report has been published regarding complete failure after the LARS procedure; however, numerous observational studies have shown excellent patient-reported outcome measures (PROMs) and generally satisfactory medium-term radiographic reduction [5, 6]. We present two cases of total LARS failure, in otherwise unremarkable patients and operations, requiring re-do repair.

## 2. Case Report

**Figure 1:**
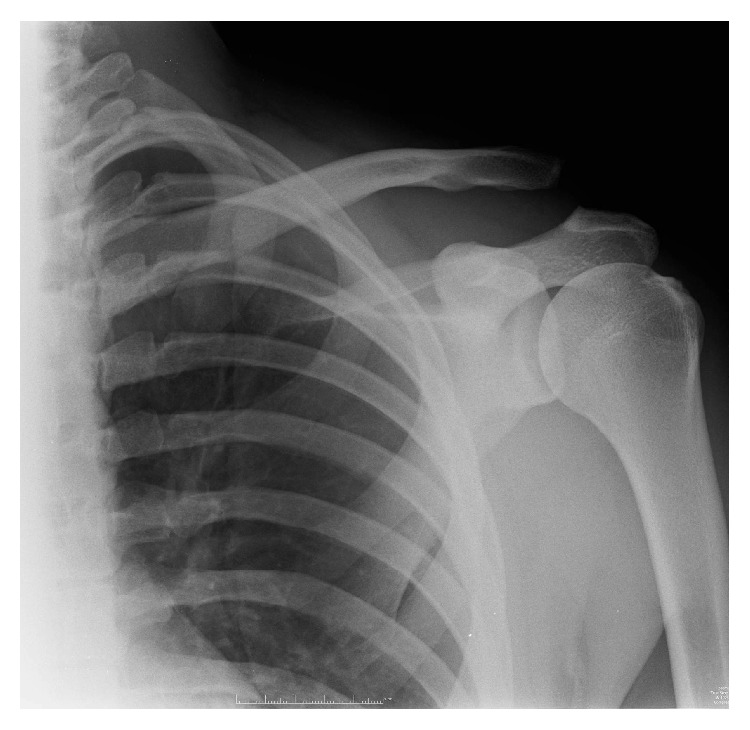
Anteroposterior left ACJ radiograph demonstrating Rockwood V ACJ dislocation.

A normally fit and well right hand dominant twenty-six-year-old man sustained a left Rockwood grade V acromioclavicular joint dislocation from a rugby injury in June 2015, complaining of pain and a grinding sensation from his left shoulder on abduction when seen in January 2016.  Figure 1 represents the preimplant radiographs. After discussion, he elected to undergo surgery to correct the position of his distal clavicle.

**Figure 2:**
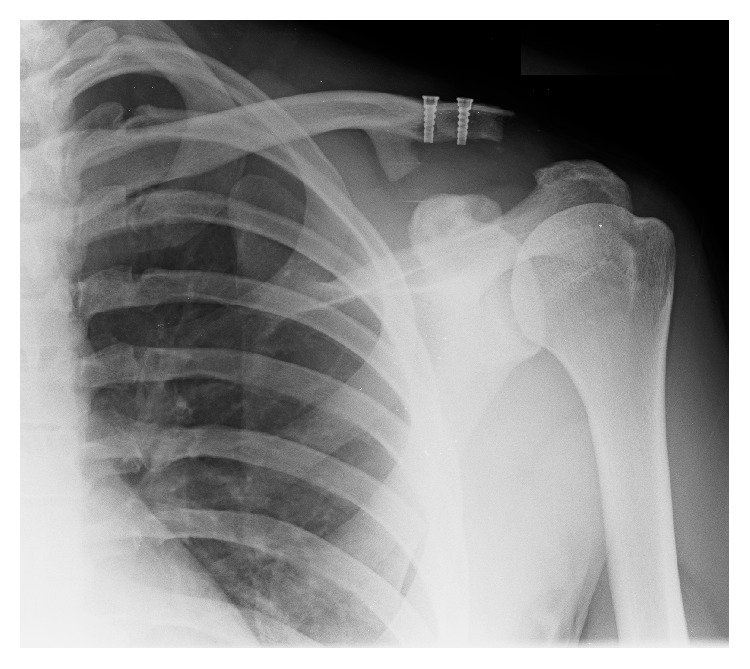
Anteroposterior left ACJ radiograph at three months after LARS July 2016 implantation demonstrating relapse of ACJ dislocation and heterotopic ossification medial to the medial clavicular screw.

An uncomplicated LARS procedure was performed in March 2016 with satisfactory intraoperative reduction and reasonable postprocedure radiographs. However, three months after operation, he re-presented with an acute history of the distal clavicle again becoming prominent and elevated with no intervening history of trauma, and subsequent radiographs demonstrated the loss of position, as seen in Figure 2. He opted for a revision procedure, performed in July 2016.

**Figure 3:**
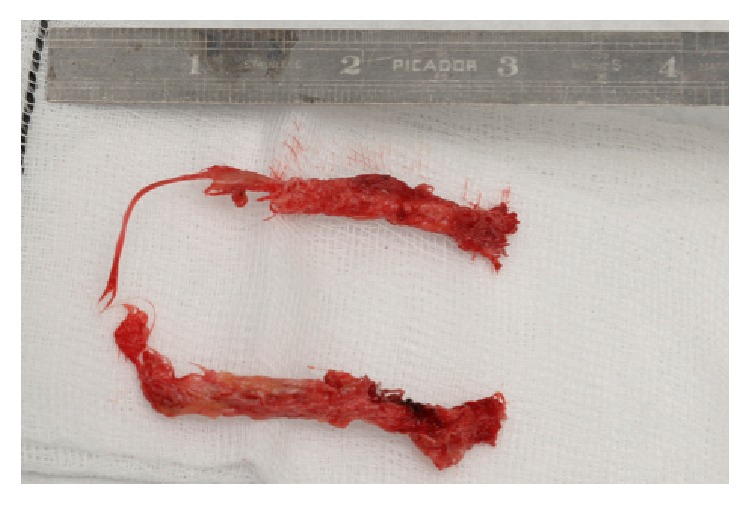
Intraoperative photograph demonstrating substantial fraying to LARS four months after insertion.

**Figure 4:**
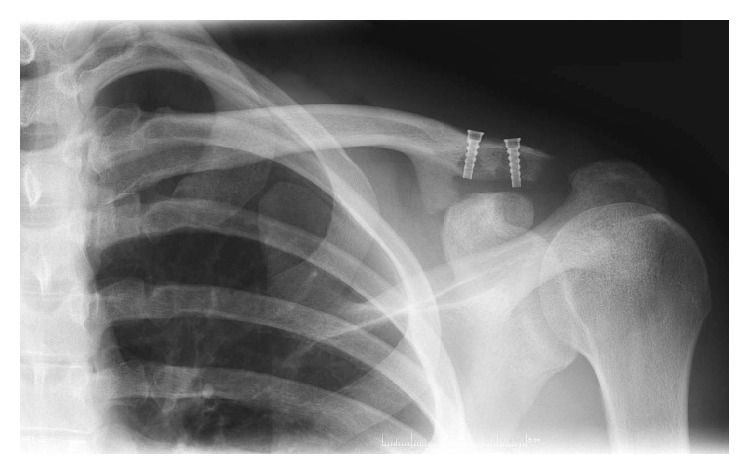
Anteroposterior left ACJ radiograph two months after re-do LARS implantation showing anatomically reduced ACJ.

Intraoperatively, the clavicular screws were secure.  Figure 3 represents the removed LARS intraoperatively. On removal, the ligament was found to be tightly held within the bone canal (on the right in Figure 3; however, the LARS was found to be frayed deep to the coracoid, as seen on the left in Figure 3). There was no apparent cause for the LARS to fray under the neck of the coracoid, as no sharp bony prominence was found that could have eroded the LARS. A revision double LARS ligament repair was completed uneventfully. The patient was seen in clinic in August 2016. Radiographs, as seen in Figure 4, demonstrated satisfactory reduction and the patient reported good function. The patient remained satisfied with the clinical outcome at repeat follow-up in September 2016 with full range of motion in the shoulder and radiographs showing a slight superior clavicular migration of 0.5 cm. Heterotopic ossification medial to the medial clavicular screw was visualized on postoperative Figures 2 and 4 but not identified intraoperatively and was considered to not be implicated in the failure.

**Figure 5:**
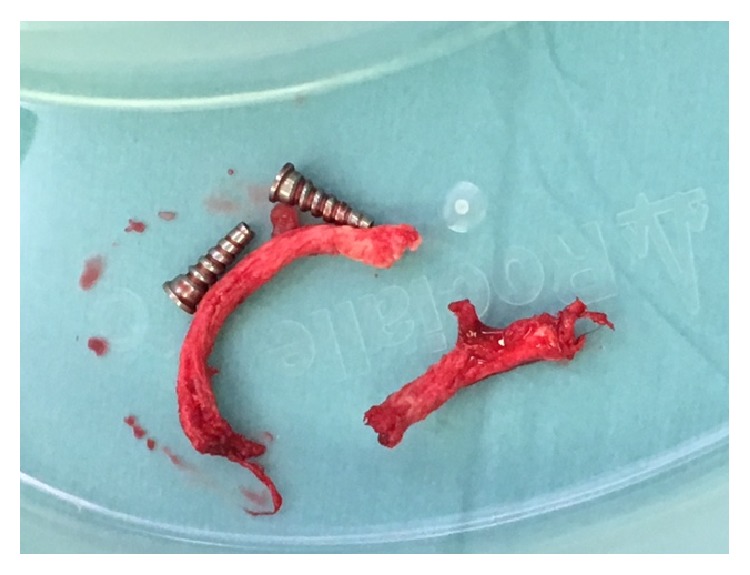
Intraoperative photograph demonstrating substantial fraying to LARS three weeks after insertion. The left fragment is the lateral limb of the LARS and the right fragment is the medial limb.

The second patient is a forty-seven-year-old male builder who had sustained a grade V Rockwood ACJ dislocation after falling from a motorbike. The ACJ dislocation was successfully reduced using LARS. The ligament seemed to have failed spontaneously, when three weeks after surgery he awoke with the distal clavicle elevated. On exploration, the LARS had frayed at its medial limb, as seen in Figure 5, between its position at the clavicle and coracoid.

## 3. Discussion

We are unaware of any studies reporting complete failure with ACJ LARS. Numerous prospective studies report excellent clinical and radiological outcomes, making these cases highly unusual. In a prospective cohort study comparing LARS PROMs between professional and nonprofessional athletes, Muccioli et al. (2016) found that 21% of patients showed slight loss of ACJ reduction, defined as a ratio between ACJ height and inferior-superior clavicular migration height, of 0.25–0.50, with no complete failures [7]. In seventeen LARS patients, Gianotti et al. (2013) found no redislocation at midterm follow-up, defined as between 1 and 41 months, and clavicular migration of only 0.1 cm postoperatively [5]. In a cohort of twenty patients treated with LARS, Fialka et al. (2000) found no functional deterioration at one-year follow-up and three cases of ACJ loosening under 5 mm, though their observations only include Rockwood III and IV ACJ dislocations [8].

LARS employs Dacron (polyethylene terephthalate), utilized in vascular implants and anterior cruciate ligament (ACL) repairs. J. Goldberg et al. (1987) followed nine patients after coracoclavicular ligament reconstruction using Dacron by sling technique and found excellent clinical, radiographic, and functional results [9].

LARS failure rates in ACL reconstructions are of the order of 2.5% and are generally attributed to technical error in tunnel placement rather than prosthetic rupture [10]. Prescott et al. (1994) found granulomatous infiltrates with marked gaps in the Dacron matrix on failed ACL Dacron specimens, but only after eight to twenty months after implant, suggesting that this ACJ LARS failure, of the order of three months, may be mechanical in origin as opposed to inflammatory [11]. The microscopic visualization of Dacron fragments in phagocytes indicated intraoperative fragmentation predisposing to chronic inflammation. Intraoperative microshredding may have occurred intraoperatively in this case and mechanistically contributed.

Whilst autologous grafting with semitendinosus or tibialis anterior tendon transfers seem to fail by rupture [12] synthetic reconstructions fail by means related to their mechanisms of action; for instance, in a prospective study three of nine patients possessing the TightRope prosthesis (Arthrex comp, Naples, FL) failed and required revision due to excessive twisting [13]. There is no indication that this occurred.

The operating surgeon considered various aetiologies for redislocation, including frayed LARS, LARS slippage through clavicular screws, medialisation of the clavicular tunnels [14] and occult clavicular fracture, though on reflection the initial operation proceeded uneventfully and the materials were intact prior to insertion. The heterotopic ossification seen on the radiographs was not found on revision surgery and was deemed unrelated to failure. The patients were compliant with postoperative instructions.

There is little literature regarding operative treatment of choice in synthetic coracoclavicular ligament failure revision, reflecting the difficulties in comparing multiple treatment options. However, Fauci et al. (2013) separated forty chronic ACJ dislocation patients into equal biological (semitendinosus allograft) and synthetic (LARS) anatomical repair cohorts and found that biological implanted patients gave statistically significantly higher Constant-Murley scores at one (88 ± 10 versus 59 ± 7.9; *p* = 0.0092) but not at four (94.2 ± 4.9 versus 85.9 ± 16; *p* = 0.0626) years of follow-up [15]. The group suggested that biological grafts provided a valuable salvage option in redislocation. Arguably the most feared complication for patient failing reconstruction already is a repeat failure. Of twenty biological patients, one redislocation occurred, as opposed to two redislocations in twenty synthetic patients, with four subluxations in each cohort. No statistical significance was calculated. The patients and authors opted for re-do LARS, as the patient was hesitant to accept the morbidity associated with autograft harvest, the similar failure rates between biologics and synthetics [15], and the opinion that recurrent failure was unlikely to occur.

Of note is that we have been using this system for over ten years with no previous failures, but this year has had another unexplained failure with a similar history, although the patient declined further surgery and so the nature of the failure could not be determined.

## 4. Conclusion

LARS failure represents a rare but functionally disappointing outcome for ACJ repair. Follow-up PROMs and radiographs for LARS reconstructions indicate very satisfactory outcomes, making these early failures highly unusual. The authors acknowledge that no method of coracoclavicular ligament reconstruction is without failure, despite being concerned by this mechanism of failure. We welcome other reports of failure in this way.
